# Refractory Fulminant Colitis Requiring Surgical Intervention in a Patient With Ulcerative Colitis on Atezolizumab Therapy for Small Cell Lung Cancer: An Atypical Case

**DOI:** 10.7759/cureus.25437

**Published:** 2022-05-29

**Authors:** Stephanie Steiger, Vincent Marcucci, Vidisha Desai, Min Zheng, Glenn Parker

**Affiliations:** 1 Surgery, St. George's University School of Medicine, St. George's, GRD; 2 Surgery, Jersey Shore University Medical Center, Neptune, USA; 3 Pathology and Laboratory Medicine, Jersey Shore University Medical Center, Neptune, USA

**Keywords:** total colectomy, programmed death ligand-1, small-cell lung cancer, atezolizumab, ulcerative colitis (uc)

## Abstract

Atezolizumab is a programmed death-ligand 1 (PD-L1) targeted antibody that prevents the binding of PD-L1 to specific T-cell receptors, thereby increasing anticancer immunity. It has been regarded as a useful first-line treatment in patients with small-cell lung cancer with a more tolerable side effect profile than chemotherapeutic agents. However, few studies focusing on the severity of adverse effects from immune checkpoint inhibitors (ICPI) have been previously reported, particularly acute fulminant colitis requiring surgical invention. We report a case of fulminant colitis refractory to high dose corticosteroid treatment in a patient with known ulcerative colitis (UC) undergoing treatment for small-cell lung cancer (SCLC) with atezolizumab. The upregulation of PD-L1 expression in patients with ulcerative colitis may play a significant role in an imbalanced T-helper cell response creating a pro-inflammatory state. The use of ICPIs to treat SCLC has been reported to increase the risk of developing inflammatory colitis. Atezolizumab use in a patient with known inflammatory bowel disease (IBD) may predispose this population to a higher risk of developing severe inflammatory colitis. We present an unusual complication associated with medical intervention in an immunocompromised patient without an established pathophysiology. The suspicion of using ICPIs in patients with IBD as a potential cause for the development of fulminant colitis is relevant and essential in the diagnostic workup for this patient population complaining of significant gastrointestinal symptoms.

## Introduction

Atezolizumab is an IgG monoclonal antibody used to treat several different cancers including breast, bladder, and hepatocellular carcinomas, as well as, small cell and non-small cell lung cancer [1]. This antibody functions by selectively binding to programmed cell death-ligand 1 (PD-L1) enhancing the tumor-specific T-cell response to promote stronger anti-tumor activity [1,2]. Immunotherapy with atezolizumab PD-L1 has produced beneficial outcomes in regard to therapeutic efficacy and in rare instances, has previously been linked to the development of acute colitis [3]. Other medications along with immune checkpoint inhibitors such as non-steroidal anti-inflammatory drugs (NSAIDs), corticosteroids, gold compounds, diuretics, tricyclic antidepressants, and neuroleptics have also been known to cause drug-induced colitis [4]. Here, we focus on checkpoint inhibitors such as anti-cytotoxic T-lymphocyte-associated antigen 4 (CTLA-4), anti-programmed cell death-1 receptor (PD-1), and its ligand (PD-L1). The most common adverse effect of these drugs is diarrhea, occurring in up to 30% of patients. However, there is only a 2% reported incidence of colitis occurring secondary to immunotherapy. This incidence increases to 12% in patients treated with combined therapy [5]. It is imperative for clinicians to understand the potential gastro-intestinal insult(s) from using immune-modulating agents, in particular, immune checkpoint inhibitors (ICPIs) such as atezolizumab, and determine the risk-benefit ratio. Herein, we present the case of a 59-year-old female with a history of ulcerative colitis (UC) and metastatic lung cancer treated with carboplatin and atezolizumab who developed pancolitis requiring surgical intervention and a pancolectomy.

## Case presentation

We report a case of a 59-year-old female with a medical history significant for poorly controlled ulcerative colitis treated with adalimumab, mesalamine, and intermittent courses of intravenous (IV) corticosteroids with poor success and a prolonged history of tobacco dependence with diagnosed chronic obstructive pulmonary disease (COPD). The patient was subsequently diagnosed with metastatic small-cell lung cancer (SCLC) with bone metastasis, confirmed by endoscopic ultrasound/fine needle aspiration and positive emission tomography (PET) scan after complaining of several weeks of worsening cough, shortness of breath, malaise, and an unintentional 15 lbs weight loss.

The patient completed five cycles of chemotherapy with carboplatin, etoposide, and infliximab over a three-month course, with no further progression of her disease process. The patient’s UC was controlled at this time and she was subsequently started on a twelve-week course of atezolizumab. She developed acute onset of generalized abdominal pain and hematochezia approximately four weeks into her twelve-week course of atezolizumab and was started on vedolizumab with resolution of her hematochezia. At the conclusion of twelve weeks, the patient was complaining of worsening generalized abdominal pain. At this time, she was started on infliximab with no resolution of her symptoms and ultimately underwent a colonoscopy with biopsy confirmed moderate pancolitis characterized by erosions, erythema, granularity, and shallow ulcerations from the anus to the cecum. The patient developed bloody bowel movements and her abdominal discomfort continued to worsen post procedure and was started on oral prednisone with no symptom improvement and she presented to the emergency department (ED) at our hospital.

**Figure 1:**
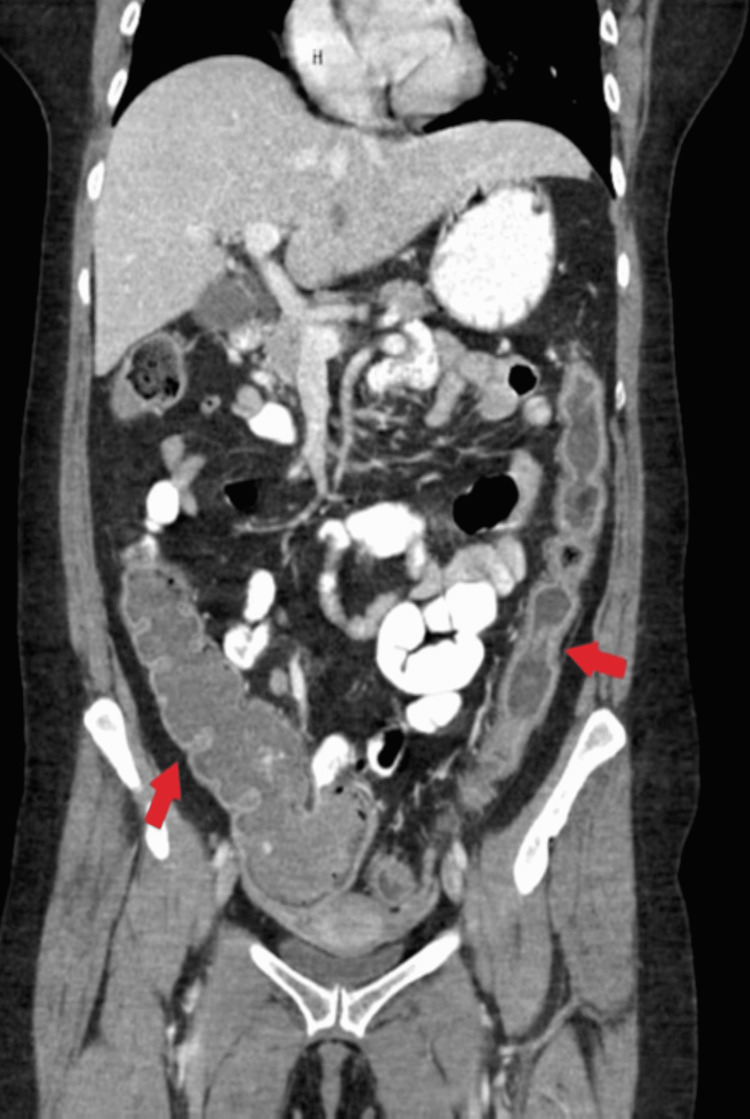
Coronal view moderate pancolitis. Red arrows indicate significant bowel wall thickening to the cecum, ascending, and descending colon

Upon presentation to the ED her hemoglobin (Hgb) was within normal limits at 12.9 g/dL. A CT of the abdomen/pelvis with PO contrast showed pancolitis as demonstrated in Figure 1. The patient refused further medical management for her gastrointestinal symptoms and requested surgical intervention. She completed an oral prednisone taper and was optimized for surgery to treat her colitis.

**Figure 2:**
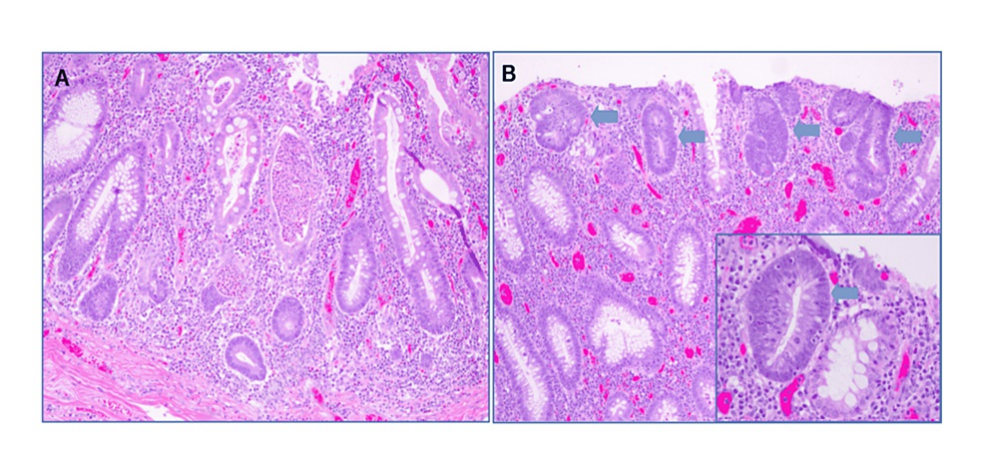
Ulcerative colitis-associated low-grade dysplasia. (A) Severe ulcerative colitis with marked acute inflammatory activity, crypt distortion, and reactive crypt epithelial change. (B) Low-grade dysplasia (arrows) involves the surface, indicating lack of surface maturation. The dysplastic gland (arrow in the inset) has epithelial cells showing pseudostratified, crowded, elongated and hyperchromatic nuclei, compared to an adjacent non-dysplastic gland. The nuclear polarity is preserved.

The patient underwent an exploratory laparotomy, total abdominal colectomy, and end ileostomy creation. Intraoperative pathology specimens were positive for chronic, active mucosal inflammation, acute cryptitis and crypt abscesses, and reactive/regenerative glandular epithelial changes shown in Figure 2. No transmural inflammation or granuloma(s) were identified. Two separate foci of mucosal low-grade dysplasia were identified in the transverse colon, manifested as nuclear crowding, chromatin hyperchromasia, and increased mitotic activity involving the mucosal surface displayed in Figure 2. No loss of nuclear polarity or complex glandular structure was identified.

## Discussion

Immunotherapy using ICPIs is becoming more common to treat solid tumor cancers [1]. There is evidence of significantly prolonged survival and progression-free survival when using atezolizumab with chemotherapy as first-line treatment in patients with diagnosed SCLC [6]. ICPIs such as atezolizumab function by targeting and binding to PD-L1 expressed on tumor cells preventing the binding of programmed death-1 (PD-1) proteins and CD80 receptors (B7-1R) located on circulating T-lymphocytes [1]. This mechanism reduces the inhibitory effect on T-lymphocyte activation against highly tumorigenic cells [1,7].

While atezolizumab and similar ICPIs have less complications and a higher survival rate than chemotherapeutic treatments, it is not without immune-related adverse events (irAE) [1]. PD-L1 inhibitors can affect several different organ systems [1,8], with gastro-intestinal (GI) complaints being the most commonly reported [9]. The GI side effect profile includes abdominal pain, bloating, reduced appetite, diarrhea, rectal bleeding, and in rare cases inflammatory colitis with a reported incidence of severe symptoms in <1% of cases [1,8,10].

The mechanism(s) associated with the development of inflammatory colitis remains unclear [9]. However, previous literature has found that ICPI toxicity within the gut is mediated to some degree by intestinal bacteria resulting in dysregulation of gastro-intestinal mucosal immunity and persistent upregulation of T-lymphocytes, creating a pro-inflammatory state [9,11]. Our patient had a known history of UC, it is uncertain if there was a causal relationship with her past medical history and the development of fulminant colitis. It is important to note the pathophysiology of UC has classically shown a strong T-helper 2 (Th2) response with elevated levels of cytokines IL-5 and IL-13 [12]. In addition to these cytokines, interactions of the B7 molecules PD-L1 and/or PD-L2 with PD-1 are known to control several tolerance checkpoints that prevent autoimmunity [12]. A study conducted by Beswick et al. in 2018 verified significant increases in total PD-L1 mRNA expression of inflamed and non-inflamed colonic mucosa in UC patients when compared against healthy controls [12]. Furthermore, PD-L1 has been reported to suppress interferon-gamma (IFN-y) production by Th1 cells, promoting a stronger Th2 response [13]. This upregulation may facilitate an imbalance in Th1/Th2 responses, potentially furthering a chronic inflammatory colonic state. The interaction ICPIs have on the immunological process and their toxicity on intestinal mucosal integrity has yet to be assessed in depth.

## Conclusions

There is no previous literature on patients treated with PD-L1 inhibitors who suffered pancolitis refractory to corticosteroid therapy resulting in a pancolectomy. Whether or not the patient’s prior atezolizumab therapy contributed to colonic inflammatory injury is difficult to determine; however, she had 12 of total doses of atezolizumab. To implicate atezolizumab in the pathological process in our patient is largely speculative. Nevertheless, it is certainly within reason to consider PD-L1 inhibitors capable of producing such an acute, dramatic histological, and clinical outcome, especially when considering the mechanism of action. Our patient developed fulminant pancolitis requiring surgical intervention following 12 recent atezolizumab doses. She was treated with high-dose corticosteroids after a colonoscopy confirmed the findings of fulminant colitis. Fulminant, refractory colitis should be considered a potential complication and included in the differential diagnosis when assessing GI complaints in patients undergoing immune checkpoint inhibitor therapy. Further research is needed to determine whether ICPI therapy should be considered an absolute contraindication in patients with uncontrolled or poorly controlled colitis.
